# Emotional Intelligence and Empathy as Determinants of Professional Quality of Life: A Psychoeducational Intervention among Healthcare Professionals

**DOI:** 10.1192/j.eurpsy.2026.11824

**Published:** 2026-07-31

**Authors:** D. Manolakaki, B. K. Puri, M. Theodoratou

**Affiliations:** 1 General Hospital of Katerini, Katerini, Greece; 2 C.A.R., Cambridge, United Kingdom; 3 Social Sciences, Hellenic Open University, Patras, Greece

## Abstract

**Introduction:**

Emotional intelligence (EI) and empathy are essential competencies for healthcare professionals working in highly demanding and emotionally charged settings. Both are closely linked to the quality of professional life (QWL), influencing well-being and workplace effectiveness.

**Objectives:**

This study aimed to (1) examine relationships between EI, empathy, and QWL among medical and nursing staff, and (2) evaluate the impact of a psychoeducational intervention designed to enhance EI and empathy in relation to QWL.

**Methods:**

A mixed-methods design was employed with 42 healthcare professionals assigned to an intervention group (n = 21, Surgical Clinic staff ) or a control group (n = 21, Operating Room staff ). Emotional intelligence was assessed using the **Wong and Law Emotional Intelligence Scale (WLEIS)**, empathy with the **Toronto Empathy Questionnaire (TEQ)**, and quality of professional life with the **Professional Quality of Life Scale (ProQOL)**. These instruments were administered before and after a four-week psychoeducational intervention combining theoretical input with experiential learning (role-play, reflective dialogue, case discussions). Statistical analyses included paired and independent t-tests, correlation analyses, and estimation of effect sizes (Cohen’s d). Qualitative data were collected through structured feedback, open discussion, and thematic analysis.

**Results:**

Baseline analyses confirmed significant positive correlations among EI, empathy, and QWL. Post-intervention, empathy and QWL demonstrated slight improvements, but EI scores in the intervention group declined. This decline may reflect increased self-awareness leading to more critical self-ratings, limited intervention duration preventing consolidation of new skills, or contextual workplace stressors. Qualitative findings nonetheless highlighted enhanced emotional awareness, greater empathic engagement, and improved communication within teams.

**Conclusions:**

EI, empathy, and QWL are strongly interrelated. Although short-term training may initially lower self-reported EI, it can foster meaningful attitudinal shifts. Sustained interventions appear necessary to achieve measurable and lasting improvements.

**Disclosure of Interest:**

None Declared

